# Pure dysgerminoma of the ovary: CT and MRI features with pathological correlation in 13 tumors

**DOI:** 10.1186/s13048-020-00674-z

**Published:** 2020-06-17

**Authors:** Shuhui Zhao, Fan Sun, Lei Bao, Caiting Chu, Haiming Li, Qiufeng Yin, Wenbin Guan, Dengbin Wang

**Affiliations:** 1 Department of Radiology, Xinhua Hospital affiliated to Shanghai Jiaotong University School of Medicine, 1665 Kongjiang Road, Shanghai, 200092 China; 2 Department of Radiology, Shanghai Cancer Center, Shanghai Medical College, Fudan University, 270 Dongan Road, Shanghai, 200032 China; 3 Department of Pathology, Xinhua Hospital affiliated to Shanghai Jiaotong University School of Medicine, 1665 Kongjiang Road, Shanghai, 200092 China

**Keywords:** Ovarian tumor, Dysgermonoma, MRI, CT, ADC value

## Abstract

**Background:**

To investigate the spectrum of CT and MRI findings of dysgerminoma of the ovary.

**Methods:**

CT and MRI imaging of 12 patients with 13 histologically proven dysgerminomas of the ovary were retrospectively reviewed. Patients^,^ ages ranged from 6 ~ 27 years (mean, 17.2 years). Two observers evaluated the following CT and MRI features of the tumor by consensus: (i) location, shape, and size; (ii) attenuation, T2 signal intensity, and ADC value; (iii) patterns of contrast enhancement; (iv) presence of fibrovascular septa; (v) presence of necrosis, hemorrhage, and calcification; (vi) presence of “ovarian vascular pedicle” sign. We also noted the extent or stage of the tumors.

**Results:**

75% lesions arised in the right ovary. Bilateral ovaries were involved in one case. Tumors displayed as a purely or predominantly solid mass (mean size, 17.0 ± 7.8 cm). Ten tumors were shaped multilobulated. The mean ADC value of lesions was 0.830 ± 0.154 × 10^− 3^ mm^2^/s. Characteristic fibrovascular septa were observed in all lesions. Among them, classic septa were present in 69% lesions. They were thin, hypointense on T2WI with a linear intense enhancement indicating the blood vessels in septa. Due to the stromal edema, fibrovascular septa may become thick even amorphous in shape, hyperintense on T2WI and even low attenuation on CT with a slight enhancement except for a bright blood vessel on the edge. Massive necrosis was observed only in one lesion. Calcification was present in 3 of the 5 tumors on CT. “Ovarian vascular pedicle” sign was present in 12 lesions. Lymphadenopathy, retroperitoneal spread, and distant metastases combined with an implantation in Douglas’ cul-de-sac were present in one patient respectively.

**Conclusion:**

On CT and MR images, ovarian dysgerminoma often appears as a large solid mass. The edematous condition of characteristic fibrovascular septa can be well displayed by imaging which then can guide the radiologists to make an accurate diagnosis. Calcifications often occur in the tumor. Nonspecific low ADC value and “ovarian vascular pedicle” sign may narrow the differential diagnosis.

## Background

Ovarian dysgerminoma, the female counterpart of testicular seminoma, derives from primitive germ cell. It is the most common ovarian malignant ovarian germ cell tumor, accounting for 32.8–37.5% of them followed by immature teratomas, yolk sac tumors, and mixed germ cell tumors [1, 2]. With a low incidence, Dysgerminoma usually occurs in the 2nd and 3rd decades of life, but 10% of cases occur in the 1st decade of life [3]. Bilateral ovaries are involved in up to 10 ~ 15% cases [3]. A palpable abdominal or pelvic mass is the most common presenting symptom, followed by abdominal pain, abdominal distention and menstrual irregularity [3]. Ovarian dysgerminoma may be associated with dysgenetic gonads that contain gonadoblastoma [2, 3]. Serum lactic dehydrogenase (LDH) is often nonspecifically elevated in patients. Serum alkaline phosphatase (ALP), CA125 and β-HCG may also be elevated, but AFP is not elevated in patients with pure dysgerminomas [3, 4]. Up to 50 ~ 75% of patients with ovarian dysgerminoma are diagnosed at stage I [4]. The treatment of patients with ovarian dysgerminoma is primarily surgical excision. Ovarian dysgerminoma is sensitive to radiotherapy and chemotherapy. Considering childbearing demands of the young patients, radiotherapy is seldom used to treat patients with ovarian dysgerminoma recently. Patients with ovarian dysgerminomas have an excellent prognosis with a 5-year survival rate of almost 100% [2, 5, 6].

The reproductive outcomes of young ovarian dysgerminoma survivors are promising with fertility-sparing treatment [7]. Preoperative imaging would play a reference role in surgical decision-making to distinguish dysgerminoma from a great variety of adnexal masses. Computed tomography (CT) is widely used in diagnosing female pelvic bulk masses for the advantages of large field of vision (FOV), reconstruction techniques and demonstration of the feeding vessels of the tumors. With the advantages of no ionizing radiation, multi-parameter imaging and high resolution of soft tissue, magnetic resonance (MR) imaging is superior in characterizing ovarian masses. As we know, there are few literatures on the imaging characteristics of ovarian dysgerminoma. Tanaka et al. studied 3 cases and reported that the characteristic imaging appearance of dysgerminoma is a multilobulated solid mass with prominent fibrovascular septa. Because of their fibrous content, the septa appear as hypointense lines on T2-weighted MR images and may show intense enhancement on contrast-enhanced CT and MR images [8]. However, accurate diagnosis of ovarian dysgerminoma remains a challenge for radiologists today. In this study, we investigated the spectrum of CT and MRI appearances of 13 ovarian dysgerminomas in 12 patients with the purpose to improve the understanding of this entity comprehensively.

## Methods

### Patient population

The institutional review board of our hospitals approved this retrospective study, and informed consent was obtained from each patient for academic use of their clinical data. Patients with suspected ovarian tumors were enrolled in an imaging study project from January 2012 to November 2019 at Xinhua Hospital affiliated to Shanghai Jiaotong University School of Medicine and Obstetrics & Gynecology Hospital of Fudan University. We found a total of 12 patients with 13 primary ovarian dysgerminomas. Patients’ ages ranged from 6 to 27 years (mean, 17.2 years). Patients presented with a palpable abdominal or pelvic mass (50%), abdominal distention (10%), abdominal pain (10%) or menstrual irregularity (30%). Gonadal dysgenesis was not found in our case series. The clinical data are listed in Table 1. Two patients underwent both CT scan and MR examination. Three patients underwent CT scan exclusively. Seven patients underwent MR examination exclusively.

**Table 1:** Clinical Data of the 12 Patients with Ovarian Dysgerminomas

Clinical features	
No. of cases (tumors)	12 (13)
Patients^,^ age (mean)	6 ~ 27 (17.2) years
Tumor diameter range (mean)	8.6 ~ 32.7 (17.9) cm
Tumor markers	
Elevated LDH (> 245 U/ml)	4/4^a^
Elevated ALP (> 135 U/ml)	3/4^a^
Elevated CA125 (> 35 U/ml)	6/8^a^
Elevated β-hCG (>3mIU/ml)	4/4^a^
Elevated AFP (> 25 ng/ml)	0/3^a^
Laterality	
Unilateral(R/L)	11 (9/2)
Bilateral	1
FIGO staging	
Stage I	9
Stage II	0
Stage III	2
Stage IV	1

^a^number of patients with the level of serum tumor marker available

### CT technique

CT examinations were performed using a 64-detector-row scanner (Brillance, Philips Medical Systems, Cleveland, OH, USA). After plain scanning, patients received intravenous bolus injection of contrast medium (Lohexol, Omnipaque; GE Healthcare, Shanghai, China) at a rate of 3 ml/sec and a dose of 2 ml/kg of body weight. Images were acquired at 30s, 60s, and 90s after the administration of contrast medium. The section thickness of all images of the signal spiral CT was 5 mm. Multiplanar reconstructions (MPR), maximum intensity projection (MIP) and volume reconstructions (VR) were conducted on the post-processing workstation.

### MR technique

MRI was performed with a 3.0 Tesla (T) MR superconductor unit (Twinspeed, GE Medical Systems, Milwaukee, WI, USA). A pelvic phased-array coil was used in each case. The patients lay in the supine position and breathed normally. The unenhanced sequences were obtained as follows: Axial T1-weighted imaging (T1WI) [time of repetition (TR) / time of echo (TE), 340 ms/10 ms]; Axial Fast spin echo (FSE) T2-weighted imaging (T2WI) with fat saturation (TR/TE, 8000 ms/83 ms); Sagittal FSE T2WI (TR/TE, 8000 ms /98 ms). An axial diffusion-weighted imaging (DWI) was performed with a b value of 1000 s/mm^2^ (TR/TE, 3350 ms/ 67.6 ms). Apparent diffusion coefficient (ADC) maps were generated automatically. The contrast-enhanced T1WI LAVA 2D with fat saturation (TR/TE, 7.87 ms/2.55 ms) was performed in the axial, sagittal and coronal planes after the injection of Gadopentetate dimeglumine (Gd-DTPA, 0.1 mmol/kg of body weight, Magnevist; Bayer Schering, Guangzhou, China) injected at a rate of 2–3 mL/s. The scanning parameters were as follows: 5-mm slice thickness, 1.5-mm gap, 128 ~ 256 × 128 ~ 256 matrix, 20–25 cm × 34 cm field of view, and four excitations. The scanning range was from the inferior pubic symphysis to the renal hilum and was extended beyond the dome of tumor in the cases with huge masses.

### Image analysis

CT and MR images were analyzed by S.H.Z. and F.S., with 11 and 5 years of experience in gynecological imaging, respectively. Their interpretations were arrived at by consensus. CT and MRI features of the tumors were assessed including (i) laterality, tumor shape, and tumor size. Maximum diameter of the mass was measured; (ii) tumor attenuation on pre-contrast CT image and T2 signal intensity on MR image. The readers rated the mass as hypoattenuating (lower density than muscles), isoattenuating (similar density to muscles), or hyperattenuating (higher density than muscles). On the T2-weighted images the masses were rated as hypointense (lower signal than outer myometrium), isointense (similar signal to outer myometrium), or hyperintense (higher signal than outer myometrium); (iii) ADC value. On ADC maps, a circular region of interest (ROI) of at least 1 cm^2^ was placed at targeted areas in the solid components of tumor, by referring to conventional MR images; (iv) patterns of enhancement. On the CT and MR studies, the enhancement degree were rated as slight (weaker than muscles), moderate (between muscles and outer myometrium), or intensive (more obvious than outer myometrium); (v) presence of fibrovascular septa. T2 signal intensity, attenuation, and enhancement degree of the septa were also recorded; (vi) presence of necrosis, hemorrhage, cystic change, and calcification in the tumor. Intratumoral cyst smaller than 2 cm in diameter was defined as small cystic space, otherwise it come to large cystic space; (vii) presence of “ovarian vascular pedicle” sign (ovarian vein extending to the mass); (viii) presence of hydronephrosis; (ix) presence of ascites. The amount of ascites was classified as small (limited to Douglas’ cul-de-sac), moderate (above level of the uterine fundus), and large (abdominal distention due to ascites). Extent or stage of the tumor was also assessed. Lymphadenopathy was considered positive if the short axis of the lymph node was larger than 6 mm in pelvic, retroperitoneal, or mesenteric region.

## Results

Twelve patients had 13 tumors in total. 11 cases occurred unilaterally including 9 cases in the right ovary and 2 cases in the left ovary. The remaining one case involved bilateral ovaries with a larger mass in the right ovary. CT and MRI features of the 13 tumors are listed in Tables 2 and 3. All 13 tumors showed as a well-encapsulated purely or predominantly solid mass. The mean size of tumors was 17.0 ± 7.8 cm with a maximum diameter exceeding 10 cm in 10 lesions. Ten tumors were multilobulated, and the other 3 tumors were round in shape.

**Table 2:** CT and MRI features of the 13 ovarian dysgerminomas in 12 patients

Case No.	Patients^,^ age (years)	Modality	Laterality	Shape	Size (cm)	Attenuation	T2 signal	ADC value (×10^−3^ mm^2^/s)	Enhancement
1	6	CT	R	Lobular	12.7	Iso-			++
2	7	CT	R	Lobular	9.4	Hypo-			+
3	10	MRI	L	Lobular	11.0		Hyper-		++
4	13	CT&MRI	R	Lobular	10.2	Iso-	Iso-	0.80	+
5	14	MRI	R	Lobular	22.1		Hyper-	0.69	+++
6	18	MRI	R	Lobular	23.2		Iso-	0.809	++
7	19	CT	R	Lobular	32.7	Iso-			+
8^a^	21	CT&MRI	r	Round	20.0	Iso-	Hyper-	1.06	++
			l	Round	8.6	Iso-	Hyper-	1.03	++
9	23	MRI	R	Lobular	20.7		Hyper-	0.714	+++
10	24	MRI	R	Round	19.2		Hyper-	1.03	++
11	24	MRI	L	Lobular	8.6		Iso-	0.709	++
12	27	MRI	R	Lobular	13.0		Hypo-		–

^a^case 8 involved bilateral ovaries

+ indicates slight enhancement; ++ indicates moderate enhancement; +++ indicates intense enhancement

**Table 3:** CT and MRI features of the 13 ovarian dysgerminomas

Case	Modality	Laterality	Fibrovascular septa types				Cystic	Hemorrhage	Calcification
No.			I	II	III	IV	change		
1	CT	R	+		+		–	–	+++
2	CT	R	+				–	–	+
3	MRI	L		+			++	+	
4	CT&MRI	R	+	+	+		–	–	+
5	MRI	R	+			+	++	+	
6	MRI	R	+	+	+		–	–	
7	CT	R	+			+	++	–	–
8^a^	CT&MRI	r				+	+	–	–
		l	+					–	–
9	MRI	R	+			+	+	+	
10	MRI	R	+			+	+	–	
11	MRI	L		+			–	–	
12	MRI	R			+		–	++	

^a^case 8 involved bilateral ovaries

+ indicates that data was positive,-indicates that data was negative

Homogenous attenuation or signal intensity was observed in the 5 relatively small masses. Heterogenous attenuation or signal intensity were seen in the 6 largest masses and 2 relatively small masses. On pre-contrast CT images, the masses had an attenuation similar to muscles. On T2-weighted images, 6 of the 10 tumors were hyperintense, 3 were isointense, and 1 was hypointense. All of the 10 lesions showed obvious high signal intensity on DW imaging (b = 1000s/mm^2^). The mean ADC value of the lesions was low to 0.830 ± 0.154 × 10^− 3^ mm^2^/s. After administration of contrast media agent, 2 lesions had an intense enhancement, 10 lesions had a moderate enhancement, and one lesion had no enhancement because of extensive necrosis.

**Fig. 1:**
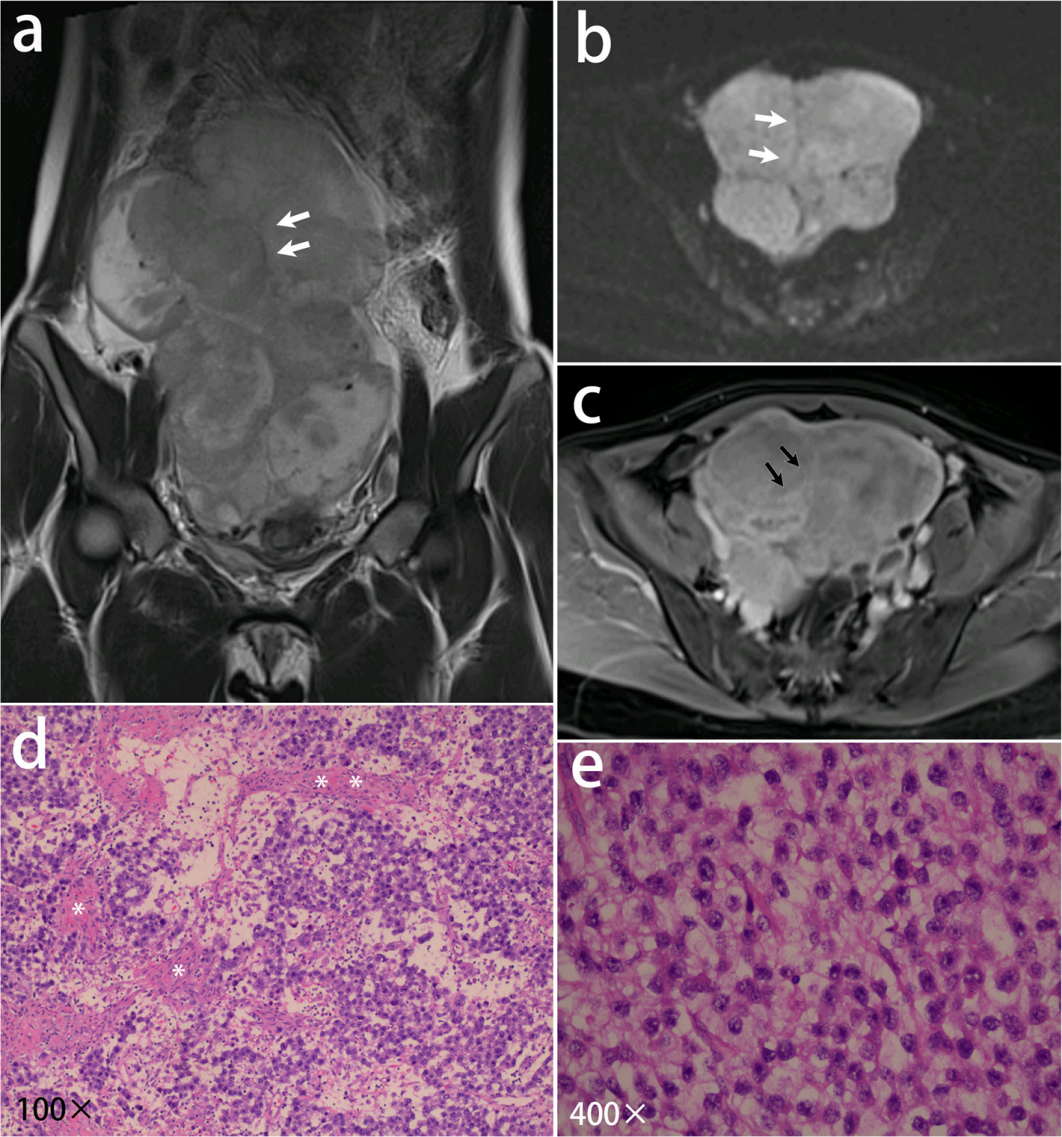
A 23-year-old woman with dysgerminoma in the right ovary. **a** Coronal T2- weighted image displays a large multilobulated heterogeneous mass with thin, hypointense septa (arrows). **b** The mass shows high signal intensity with the septa as low signal intensity (arrows) on DW imaging (b = 1000 s/mm^2^). **c** The mass has a moderate contrast enhancement on T1-weighted images. The septa are enhanced obviously (arrows). **d** The photomicrograph (H&E, 100×) shows tumor cells arranged in islands separated by fibrous septa (*). **e** Higher power image (H&E, 400×) shows polygonal tumor cells with distinct cell borders

**Fig. 2:**
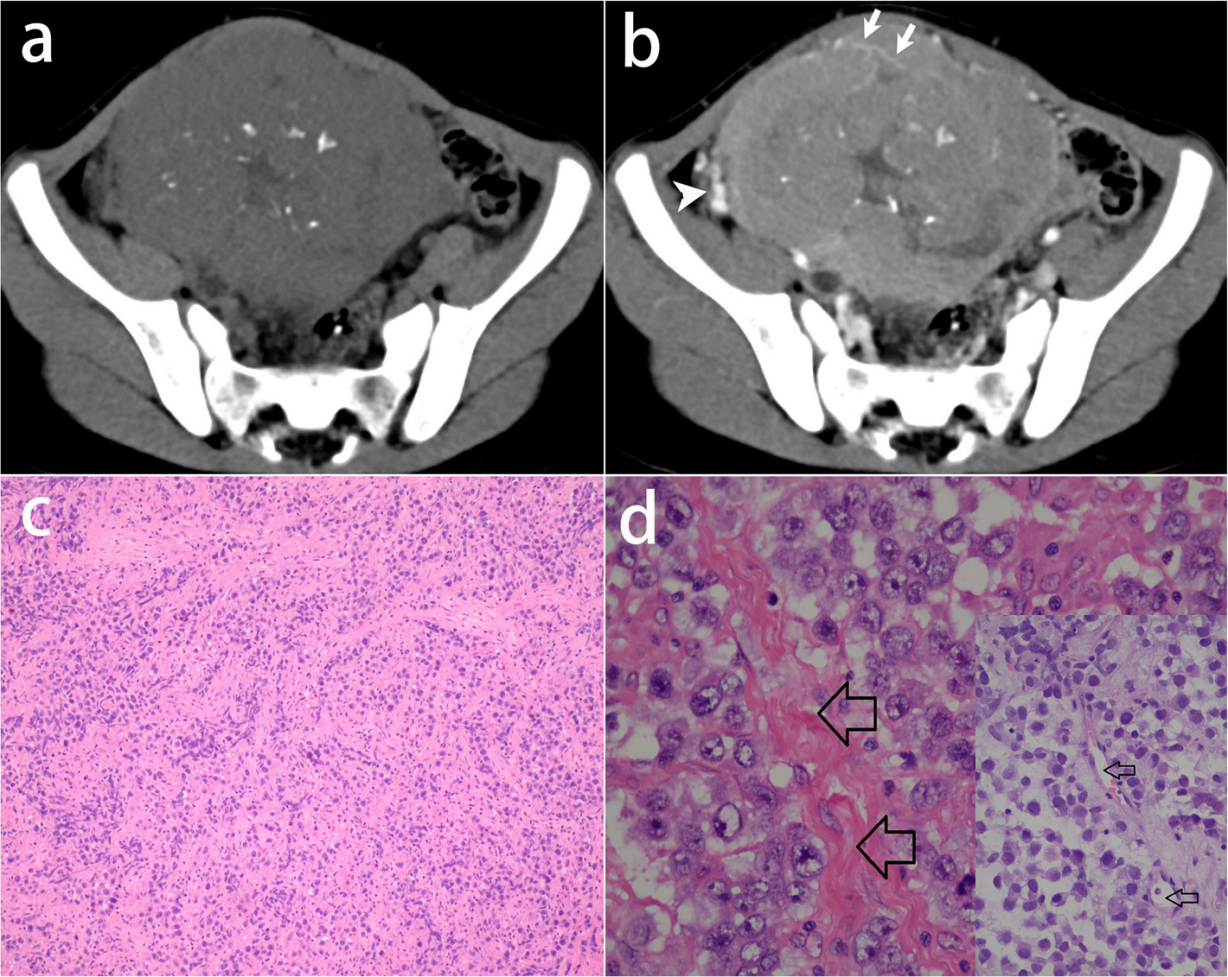
A 6-year-old girl with dysgerminoma in the right ovary. **a** Pre-contrast CT image shows a solid mass with speckled calcifications in the tumor. **b** Intratumoral linear vessels (white arrows) are recognized on post-contrast CT image. Ipsilateral ovarian vein was obviously thickened (arrowhead). **c** The photomicrograph (H&E, 100×) shows tumor cells arranged in islands separated by rich fibrous septa. **d** Images at high magnification (H&E, 400×) show the blood vessels (thin arrows) in fibrous septa (thick arrows)

**Fig. 3:**
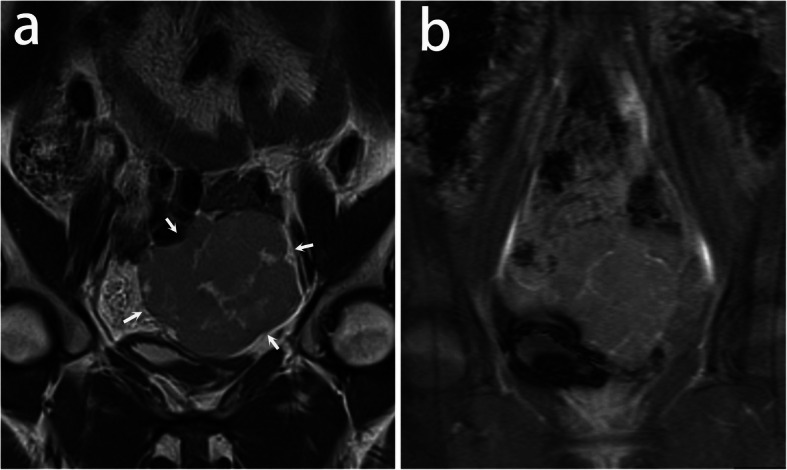
A 24-year-old woman with dysgerminoma in the left ovary. **a** Coronal T2-weighted image demonstrates a solid mass (arrows) with multiple hyperintense septa because of edema. **b** Coronal T1- weighted contrast enhanced image shows the mass has a moderate enhancement. The septa are enhanced linearly and markedly

**Fig. 4:**
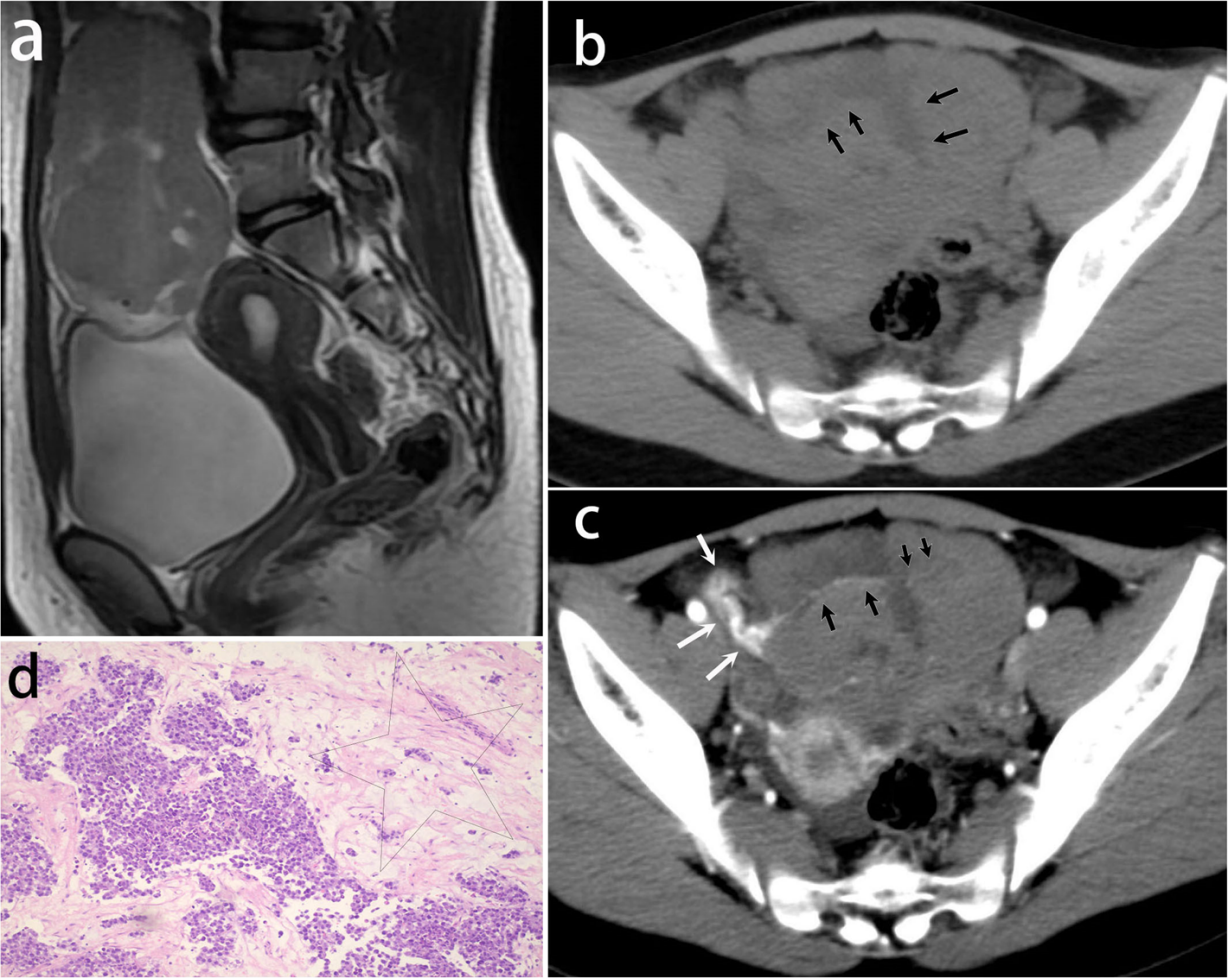
A 13-year-old girl with dysgerminoma in the right ovary. **a** Sagittal T2-weighted image shows an isointense mass with multiple hyperintense foci. **b** Thick septa (arrows) display as low density on pre-contrast CT scan. **c** The mass has a contrast enhancement similar to muscles. The septa have slight enhancement except for a linear marked enhancement on edge which indicating blood vessels in septa (black arrows). Twist ovarian vascular pedicle (white arrows) is present indicating a torsion of right adnexa. **d** The photomicrograph (H&E, 100×) shows thick edematous fibrous septa (star)

**Fig. 5:**
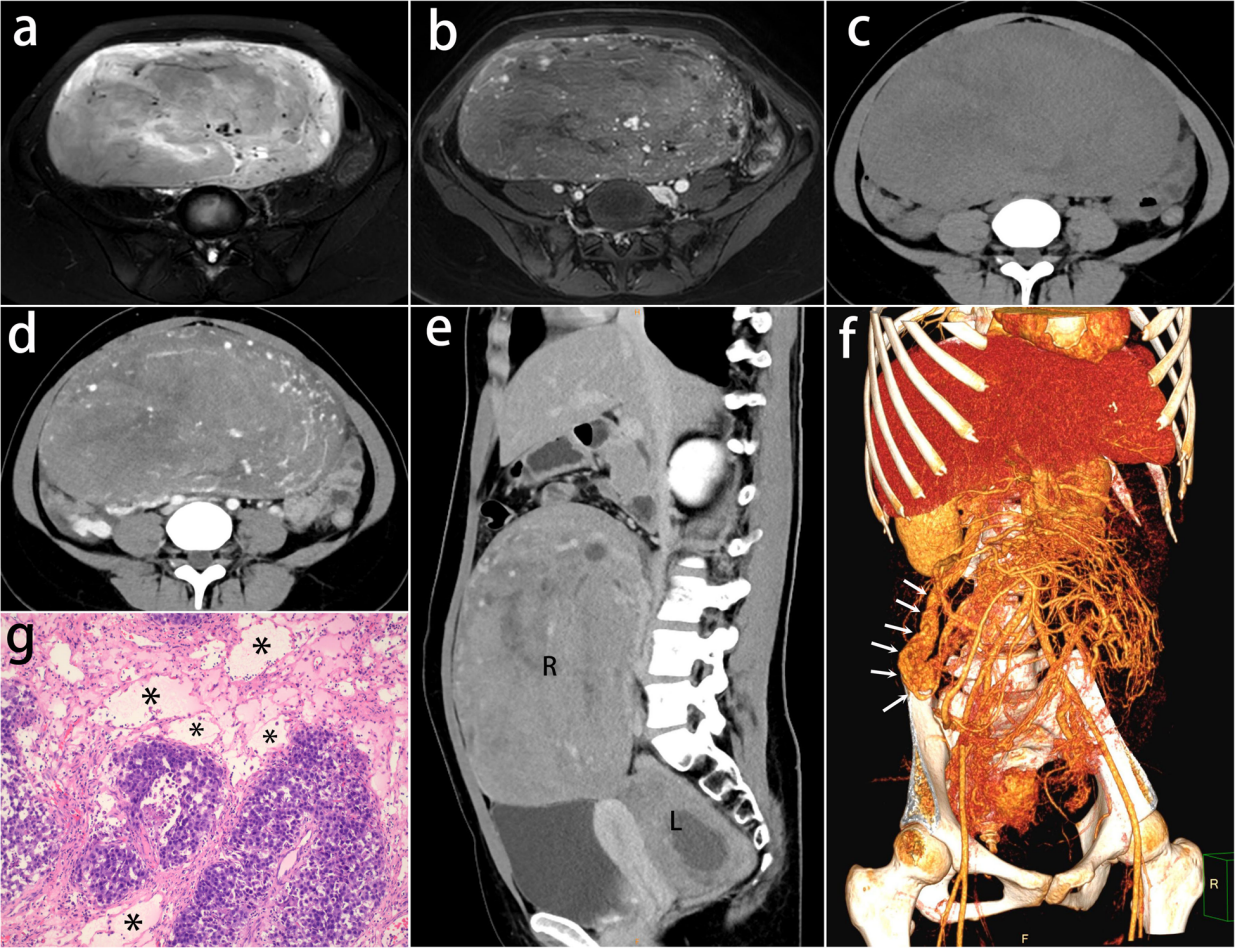
A 21-year-old woman with bilateral ovarian dysgerminomas. **a** Axial T2-weighted with fat suppression image displays a large heterogeneous solid mass. There are massive void vessels in amorphous hyperintense septa. **b** The mass has a moderate contrast enhancement on T1-weighted image. The septa are enhanced slightly and the dilated vessels are enhanced markedly. **c**, **d** Pre- and post -contrast CT scan show a heterogeneous mass with prominent intratumoral vessels. **e** Sagittal MPR displays the bilateral lesions (R and L). **f** VR demonstrates the thickened ovarian vein (arrows) and the extending intratumoral reticular vessels. **g** The photomicrograph (H&E, 100×) shows the stromal septa in the upper were edematous and amorphous with massive dilated blood vessels (*)

Characteristic fibrovascular septa were observed in all lesions. According to the degree of edema, septa were categorized into four types. Type I: thin non-edematous septa which were seen in 69% lesions. Those classic septa were hypointense on T2-weighted images, and unrecognizable on pre-contrast CT images. After administration of Gd-DTPA, it showed a linear and intense enhancement indicating the small blood vessels in septa (Figs. 1, 2); Type II: thin edematous septa which were seen in 31% lesions. Those septa were hyperintense on T2-weighted images. It had a similar contrast enhancement to type I (Fig. 3); Type III: thick edematous septa which were seen in 31% lesions. Those septa were hyperintense on T2-weighted images and hypoattenuating on pre-contrast CT images. They had a mild or no contrast enhancement except for a bright blood vessel on the edge (Fig. 4); Type IV: map-shaped edematous septa which were seen in 38% lesions. Those septa were amorphous and extensive in lesions. They were only seen in large masses (size range,19.2 ~ 32.7 cm), and always gave the tumors a heterogenous appearance. They had a similar T2 signal, attenuation, and enhancement to Type III. The blood vessels in Type IV septa were often markedly dilated (Fig. 5). In 7 of the 13 (54%) tumors, more than one types of septa co-existed in one lesion. All types of fibrovascular septa showed a low signal intensity on DW imaging.

Extensive necrosis and obvious hemorrhage was present in one lesion. The entire lesion had no enhancement but did not show cystic change. There was no vascularity observed within or in the periphery of the lesion on contrast images. Moreover, the necrosis and hemorrhage in the lesion caused a symptom of acute abdominal pain. Minor hemorrhage was present in 3 cases. The hemorrhage showed hyperintense on T1-weighted image. Small cystic space was present in 6 lesions, and large cystic space was present in 3 of the 6 lesions. On CT images, calcifications were observed in 3 of the 5 cases. The shape of calcification was speckled, linear inside the tumor and linear in the capsule respectively (Fig. 2).

“Ovarian vascular pedicle” sign was present in 12 lesions except for the one lesion with extensive necrosis. In contrast to the contralateral ovarian vein, ipsilateral ovarian vein was obviously thickened and tortuous especially in huge masses (Figs. 2, 4, 5). On T2-weighted image, ipsilateral thickened ovarian vein had an obvious flow-void effect. In addition, a twisted ovarian vascular pedicle was observed in the two cases complicated with a torsion of ipsilateral adnexa (Fig. 4).

Nine of the 12 (75%) cases were at stage I. The two cases at stage III had mesenteric root lymphadenopathy and retroperitoneal spread respectively. Calcifications were seen in the retroperitoneal spread. The one case at stage IV had implantation in Douglas’ cul-de-sac and distant metastasis to the subcutaneous tissue of abdominal wall. A large amount of ascites was present only in 2 cases with one case accompanied with pleural effusion (pseudo meigs syndrome). Hydronephrosis was present in 3 cases.

## Discussion

Ovarian dysgerminoma is the most common malignant germ cell tumor of the ovary, affecting mostly patients in the second to third decade. It has a predilection for arising in the right ovary. It is reported that the tumors were in 51% located in the right ovary, in 32% in the left ovary and 17% bilateral [9]. In current study, 75% of cases had a right-sided lesion, 16.7% of cases had a left-sided lesion and 8.3% of cases had bilateral lesions. The histogenesis of dysgerminoma may account for its predilection for the right ovary that the differentiation of ovarian tissue on the right side proceeds more slowly and to a less degree than it does on the left side [9]. In addition, ovarian dysgerminoma may be the only tumor which can occur bilaterally in all malignant germ cell tumors [3].

We found that a large well-encapsulated, multilobulated, purely or predominantly solid mass is the gross imaging appearance of ovarian dysgerminoma. It demonstrates an attenuation similar to muscles, and nonspecific signal intensity on T2-weighted image. A heterogenous attenuation or signal intensity tend to be present in large masses. Consistent with its malignancy, ovarian dysgerminoma displayed a high signal intensity on DW imaging with a mean ADC value low to 0.830 ± 0.154 × 10^− 3^ mm^2^/s. As far as we know, ADC value of ovarian dysgerminoma has not been reported in previous study. Unlike yolk sac tumor, ovarian dysgerminomas usually have an enhancement lower than myometrium on contrast-enhanced CT and MR images [10].

The characteristic imaging feature of dysgerminoma is fibrovascular septa in the tumor which had been revealed by Tanaka in 1994. Because of their fibrous content, the classic fibrovascular septa appear as hypointense lines on T2-weighted images and show intense enhancement on contrast-enhanced CT and MR images [1, 8]. In agreement with previous study, fibrovascular septa were present in all our cases on CT and (or) MR images. Besides, our study had a novel finding that due to the stromal edema, fibrovascular septa may become thickened even amorphous in shape, hyperintense on T2-weighted images and even low attenuation on CT images with a slight enhancement except for a bright blood vessel on edge. According to the degree of edema, we divided the septa into four types: thin non-edematous septa, thin edematous septa, thick edematous septa, and map-shaped edematous septa. The map-shaped edematous septa present only in huge masses. About half of cases had more than one types of septa. Obviously, the presence of stromal edema may be critical for accurate diagnosis since it can strongly affect the shape, signal intensity and attenuation of fibrovascular septa, or even the entire lesion.

Ovarian dysgerminoma may also contain necrosis, hemorrhage, small cystic change or calcifications. Unlike yolk sac tumor, massive necrosis and hemorrhage were much less common in dysgerminoma. In this study, massive necrosis was observed only in one case in which the entire lesion had no enhancement with obvious hemorrhage. We speculate the infarction may be caused by the obstruction of drainage vein at the periphery of the lesion which was indicated by the absence of “ovarian vascular pedicle” sign. In addition, we noticed calcifications were present in up to 60% lesions and even in the retroperitoneal spread. It is said that the presence of calcification suggests an underlying gonadoblastoma [11]. However gonadal dysgenesis was found in none of our patients.

It is reported that “ovarian vascular pedicle” sign was present in 92% of ovarian masses [12–14]. In the present study, it was present in 92% of dysgerminomas. Moreover, we noted that ipsilateral ovarian vein was obviously thickened and tortuous especially in huge dysgerminomas. Despite those findings are not specific to dysgerminomas, it may be a critical indicator for determination of the ovarian origin and narrow the differential diagnosis. Twisted ovarian vascular pedicle has been previously described as an important CT and MRI imaging feature of ovarian torsion [15]. In agreement with previous study, twisted ovarian vascular pedicle was also recognized in the two cases complicated with a torsion of ipsilateral adnexa in the present study.

Our study has some limitations. Firstly, due to the rarity of ovarian dysgermonoma, the sample is relatively small in this retrospective study. Prospective large sample investigation merits further study. Secondly, it was difficult to correlate the radiological finding and the gross pathology completely since some of the cases received chemotherapy before resection surgery.

## Conclusions

In conclusion, ovarian dysgermonoma has a predilection for arising in the right ovary. It often appears as a large multilobulated purely or predominantly solid mass in CT and MR imaging. The characteristic fibrovascular septa without edema are thin, T2 hypointense, and prominently enhanced. The edematous fibrovascular septa may be thick, T2 hyperintense, hypoattenuating and slightly even not enhanced except for a bright blood vessel on the edge. In addition, calcifications often occur in the tumor. Nonspecific low ADC value and “ovarian vascular pedicle” sign may narrow the differential diagnosis.

## Data Availability

Please contact the first author for data requests.

## Abbreviations

CT: Computed tomography
FOV: Field of vision
DW: Diffusion-weighted
MR: Magnetic resonance
ADC: Apparent diffusion coefficient
